# Long-Term Exposure to Ambient Air Pollution and Incident Amyotrophic Lateral Sclerosis: A Prospective Cohort Analysis of the UK Biobank

**DOI:** 10.1212/WNL.0000000000214858

**Published:** 2026-04-03

**Authors:** Christos V. Chalitsios, Oliver Rudolf, Jiali Gao, Martin R. Turner, Alexander G. Thompson

**Affiliations:** Nuffield Department of Clinical Neurosciences, University of Oxford, United Kingdom.

## Abstract

**Background and Objectives:**

Amyotrophic lateral sclerosis (ALS) is a neurodegenerative disease with a complex etiology. Although a range of genetic and lifestyle factors have been implicated, the potential role of environmental airborne pollution exposure is uncertain. This study examined the association between long-term ambient exposure to air pollutants and the incidence of ALS in UK Biobank participants.

**Methods:**

This prospective cohort study was based on the UK Biobank participants aged 40–69 years. The analytical sample comprised participants free of ALS at baseline and had complete data on air pollution exposure. Long-term exposure (2006–2021) to nitrogen dioxide (NO_2_), nitrogen oxides (NO_X_), fine particulate matter (PM_2.5_; <2.5 µm), and coarse particulate matter (PM_10_; <10 µm) was assessed using data from the UK Department for Environment, Food and Rural Affairs at a spatial resolution of 1 × 1 km. To evaluate the association between these pollutants and ALS risk, we used multivariable time-varying Cox proportional hazards models. Several sensitivity analyses were conducted to assess the robustness of the results. We also examined for gene-environment interaction stratified by *C9orf72* status and *UNC13A* genotype.

**Results:**

Among the 501,308 participants with a mean age of 56.5 (SD 8.1) years at baseline, 272,764 (54.4%) were female. Over a median follow-up of 8.4 years, 687 individuals developed ALS. We did not observe any associations for any of the examined pollutants and ALS risk. Specifically, the hazard ratios per SD increment for PM_10_, PM_2.5_, NO_X_, and NO_2_ were 1.03 (95% CI 0.92–1.15), 1.00 (95% CI 0.88–1.14), 1.01 (95% CI 0.90–1.13), and 1.00 (95% CI 0.89–1.12), respectively. Individuals living in areas with the highest tertile of air pollutant exposure, compared with those in the lowest tertile, did not show a higher risk of ALS across any of the pollutants examined (*p* for trend >0.05). Restricted cubic spline analyses revealed no nonlinear associations between air pollution and ALS risk (all *p* for nonlinearity >0.05). These results remained robust in various subgroup and sensitivity analyses. No evidence of gene-environment interaction was found.

**Discussion:**

In this large population-based study with high statistical power, ambient air pollution was not a risk factor for the development of ALS.

## Introduction

The adult-onset neurodegenerative disease amyotrophic lateral sclerosis (ALS) exemplifies a pathogenically complex disorder. Although approximately 10% of cases are attributable to penetrant monogenetic single-gene variants, the remaining majority of cases have a less defined etiology with interactions between underlying genetic susceptibility, ageing, and potentially modifiable exposures influencing disease risk.^1^ Incomplete penetrance and wide variation in age at onset in those with monogenetic ALS suggest that risk factor modification may have a role in prevention, even in those with high genetic risk.^2^ Although advances in laboratory analysis have substantially enhanced understanding of the genetic architecture of ALS,^3^ potentially modifiable factors have proven difficult to definitively identify.

Air pollution is a potentially modifiable environmental factor that has been associated with a broad spectrum of adverse health outcomes, including neurodegenerative diseases such as forms of dementia and Parkinson disease.^4-6^ It is hypothesized that ultrafine airborne particles may translocate systemically and penetrate or disrupt the blood-brain barrier, initiating chronic neuroinflammation, microglial activation, oxidative stress, and white matter abnormalities.^7-9^ Evidence regarding the association between air pollution and ALS remains limited and inconsistent. A small number of case-control studies have explored this association, with some suggesting a potential association between air pollutants and ALS, whereas others have not^10-15^ (summarized in eTable 1). In addition, these studies did not use longitudinal exposure data for air pollutants, limiting their ability to capture long-term variability and residential mobility over time.

We therefore analyzed data within a large population-based cohort, UK Biobank, to investigate the association between long-term exposure to ambient air pollutants, including longitudinal measurements of particulate matter with a diameter less than 2.5 μm (PM_2.5_) and 10 μm (PM_10_), nitrogen dioxide (NO_2_), and nitrogen oxides (NO_X_), and the incidence of ALS.

## Methods

### Data Sources

Data were acquired from the UK Biobank, with around 502,000 volunteering participants aged 40–69 years across England, Wales, and Scotland recruited between March 13, 2006, and October 1, 2010. At baseline visit, data on demographics, lifestyle, and health status were collected at 22 assessment centers through touchscreen questionnaires, verbal interviews, and physical measurements. Health-related outcomes are available through linked records from primary care, hospital inpatient, death, and cancer registers.

### Study Population

All participants who had not been diagnosed with ALS by the time of recruitment were included in this study.

Sex was defined based on genetically inferred sex (XX or XY chromosomes), which served as an enrolment criterion. We excluded individuals with aneuploidy or if there was a mismatch between the self-reported and biological sex.

### Covariate Assessment

Through interviews and questionnaires, information was collected at baseline, primarily self-reported, covering various aspects, including demographics (age, sex), socioeconomic (Townsend Deprivation Index [TDI]), lifestyle characteristics (smoking status, alcohol consumption, age completing full-time education, and employment status), anthropometric measures (body mass index [BMI]), medical history, and history of comorbidities.

### Exposure Assessment

The UK Department for Environment, Food and Rural Affairs (DEFRA) compiles comprehensive data on near-surface air pollution,^16^ which has been widely used in epidemiologic research.^4,17,18^ This open-access repository provides annual average concentrations of multiple air pollutants from 2001 to 2023 at a spatial resolution of 1 × 1 km. Concentration estimates are generated using an air dispersion modelling system that incorporates inputs from the National Atmospheric Emissions Inventory, secondary inorganic aerosol measurements, and emissions from sources such as dust resuspension. These modelled estimates are further refined through calibration with measurements from background monitoring stations in DEFRA's Automatic Urban and Rural Network. DEFRA routinely compares modelled and measured annual mean concentrations, consistently demonstrating strong concordance. Detailed model validation metrics and performance evaluations are publicly available.^19^

Following established methodologies from previous studies,^4,17,18^ we derived annual exposure estimates for PM_2.5_, PM_10_, NO_2_, and NO_X_ for each UK Biobank participant by linking modelled concentration data to their geocoded residential address histories. Address records in UK Biobank are updated through participant self-report (online or through the contact center) and periodic linkage exercises using NHS and other verified sources, ensuring that changes in residence are captured throughout follow-up. Residential mobility was incorporated, allowing exposures to be updated whenever participants changed address.

### Outcome Assessment

Incident ALS information was obtained from linked Hospital Episode Statistics^20^ or death registry data across England, Wales, and Scotland. The ICD-9 (335.2) and ICD-10 (G12.2) codes were used to define ALS diagnosis. The estimated accuracy of algorithmically defined ALS events using routine linked health records has been validated, with a positive predicted value between 55% and 92%.^21^

### Statistical Analysis

We used multivariable time-varying Cox proportional hazards models to evaluate the associations between air pollutant exposures, modelled both as continuous variables and categorized into tertiles, and the risk of ALS. The results were reported as hazard ratios (HRs) with corresponding 95% CIs. We calculated robust standard errors to account for the within-center correlation of observations across assessment centers. The proportional hazards assumption was tested using Schoenfeld residuals, with no evidence of violation. To enhance model robustness, we adjusted for a range of potential confounders, including age (as a time-varying covariate), sex, BMI, smoking status, TDI, and physical activity. To assess any potential nonlinear association between air pollutants and ALS risk, we used time-varying Cox proportional hazards models with restricted cubic splines, with knots at the 5th, 50th (reference), and 95th centiles, compared with the linear time-varying Cox model using the likelihood ratio test. We additionally conducted subgroup analyses by sex, and, to examine possible gene-environment interactions, by *C9orf72* hexanucleotide repeat expansion (HRE; >100 repeats vs <30 repeats), and by *UNC13A* rs12608932 genotype (C-allele homozygotes vs all other genotypes). *C9orf72* HRE is the most frequent rare-variant cause of ALS in European populations, and homozygosity for the C risk allele at rs12608932 in *UNC13A*, which occurs at appreciable frequency in European-ancestry populations (approximately 10%–13%), has been associated with higher ALS risk.^2^ C9orf72 HRE status was obtained using ExpansionHunter of whole-genome sequencing data, and genotype at rs12608932 of *UNC13A* was obtained based on UK Biobank array data as described in our previous work.^2^ We also evaluated potential effect modification by including interaction terms for sex, *C9orf72*, and *UNC13A* genotype in models.

We estimated statistical power using an analytical approximation for Cox proportional hazards models with continuous exposures, based on methods originally proposed by Freedman^22^ and extended by Hsieh and Lavori^23^: $\text{Power}=\Phi \left(\sqrt{d}\cdot \log\left(\text{HR}\right)\cdot \sigma -z_{1-\alpha /2}\right)$, where Φ is the cumulative distribution function of the standard normal distribution, d is the number of events, and σ is the SD of the exposure.

A series of sensitivity analyses was conducted to assess the robustness of our findings. First, we excluded participants diagnosed with ALS within the first 2, 3, 4, and 6 years of follow-up to mitigate potential reverse causation. Second, we restricted the analysis to participants who did not change residence during the follow-up period, to eliminate potential bias due to residential mobility. Third, we re-ran the analysis using only baseline air pollution measurements to evaluate the effect of time-varying exposure specification.

All analyses were performed using R software (version 4.2.0; R Foundation for Statistical Computing, Vienna, Austria). Two-sided *p* values less than 0.05 were considered statistically significant.

### Standard Protocol Approvals, Registrations, and Patient Consents

The UK Biobank received ethical approval from the research ethics committee (REC reference for UK Biobank 11/NW/0382), and participants provided written informed consent. This research was conducted using the UK Biobank Resource under application number 57629.

### Data Availability

The data that support the findings of this study are available from UK Biobank, subject to a registration and application process (see ukbiobank.ac.uk).

## Results

### Baseline Characteristics and Air Pollution Exposure

After excluding participants with prevalent ALS at baseline (n = 54) to minimize reverse causation, as well 842 individuals with aneuploidy (those with putative sex chromosome configurations other than XX or XY) or with a mismatch between self-reported and biological sex to avoid misclassification bias, the study included 501,308 participants. Among these participants, 687 developed ALS over a median follow-up time of 8.37 years (Table 1). Compared with individuals without ALS, those with ALS were older at recruitment (median age: 58 vs 62 years), were more likely to be male (55.6% vs 45.6%), and were former or current smokers (50.5% vs 44.9). BMI was similar between the groups, with mean values of 27.4 and 27.4 kg/m^2^ for individuals with and without ALS, respectively. Physical activity levels were slightly lower among ALS cases, with 16.9% reporting low activity, compared with 14.2% among those without ALS.

**Table 1:** Baseline Characteristics of ALS and Non-ALS Cohorts

	ALS events (N = 687)	Non-ALS events (N = 500,621)
Follow-up time, y		
Mean (SD)	7.99 (3.79)	13.3 (2.03)
Median (IQR)	8.37 (6.09)	13.6 (1.47)
Age at recruitment, y		
Mean (SD)	60.8 (6.59)	56.5 (8.09)
Median (IQR)	62 (8)	58 (13)
Sex		
Male	382 (55.6)	228,162 (45.6)
Female	305 (44.4)	272,459 (54.4)
Ethnicity^a^		
Non-White	21 (3.1)	26,942 (5.4)
White	660 (96.1)	470,916 (94.1)
Missing	6 (0.9)	2,763 (0.6)
BMI, kg/m^2^		
Mean (SD)	27.3 (4.53)	27.4 (4.80)
Median (IQR)	26.8 (5.54)	26.7 (5.77)
Missing	13	3,076
Smoking status		
Never	339 (49.3)	272,582 (54.4)
Previous	273 (39.97)	172,388 (34.4)
Current	72 (10.5)	52,714 (10.5)
Missing	3 (0.4)	2,937 (0.6)
Alcohol consumption frequency		
Never	53 (7.7)	40,405 (8.1)
<3 times per week	345 (50.2)	242,151 (48.4)
3+ times per week	287 (41.8)	216,571 (43.3)
Missing	2 (0.3)	1,494 (0.3)
Physical activity, IPAQ		
Low	116 (16.9)	71,284 (14.2)
Moderate	194 (28.2)	156,072 (31.2)
High	194 (28.2)	156,602 (31.3)
Missing	183 (26.6)	116,663 (23.3)
TDI		
Mean (SD)	−1.34 (3.07)	−1.30 (3.09)
Median (IQR)	−2.18 (4.08)	−2.14 (4.19)
Missing	2	622
Employment status		
Employed	269 (39.2)	286,237 (57.2)
Not employed	415 (60.4)	212,317 (42.4)
Missing	3 (0.4)	2,067 (0.4)
Self-rated overall health		
Excellent	109 (15.9)	81,609 (16.3)
Good	361 (52.5)	288,119 (57.6)
Fair	164 (23.9)	104,850 (20.9)
Poor	47 (6.8)	22,573 (4.5)
Missing	6 (0.9)	3,470 (0.7)
*C9orf72* carriers	74 (10.8)	626 (0.1)
*UNC13A rs12608932*		
CC	132 (19.2)	60,582 (12.1)
AC	270 (39.3)	221,281 (44.2)
AA	263 (38.2)	203,730 (40.7)
Missing	22 (3.2)	15,028 (3)

Abbreviations: ALS = amyotrophic lateral sclerosis; BMI = body mass index; IPAQ = International Physical Activity Questionnaire; IQR = interquartile range; TDI = Townsend Deprivation Index.

All the figures are expressed as absolute numbers (and percentages), unless otherwise specified.

a Ethnicity was derived from self-reported ethnic background collected at baseline assessment. Participants were asked the question “What is your ethnic group?” with the following response options (using standard UK census categories): White, Mixed, Asian or Asian British, Black or Black British, Chinese, Other ethnic group, Do not know, and Prefer not to answer. For this study, and due to small numbers in individual non-White categories, responses were collapsed into White and non-White for descriptive purposes only.

Mean annual concentrations of each air pollutant declined steadily over the study period (eFigure 1). The mean (SD) annual concentrations of PM_2.5_, PM_10_, NO_2_, and NO_x_ among ALS and non-ALS cohorts were (10 [2.1] vs 9.2 [2.3]), (14.6 [2.1] vs 13.8 [2.8]), (18 [6.5] vs 16.2 [6.7]), and (26.8 [12] vs 23.9 [11.9]) μg/m^3^, respectively (eTable 2).

### Air Pollutants and ALS

**Figure 1:**
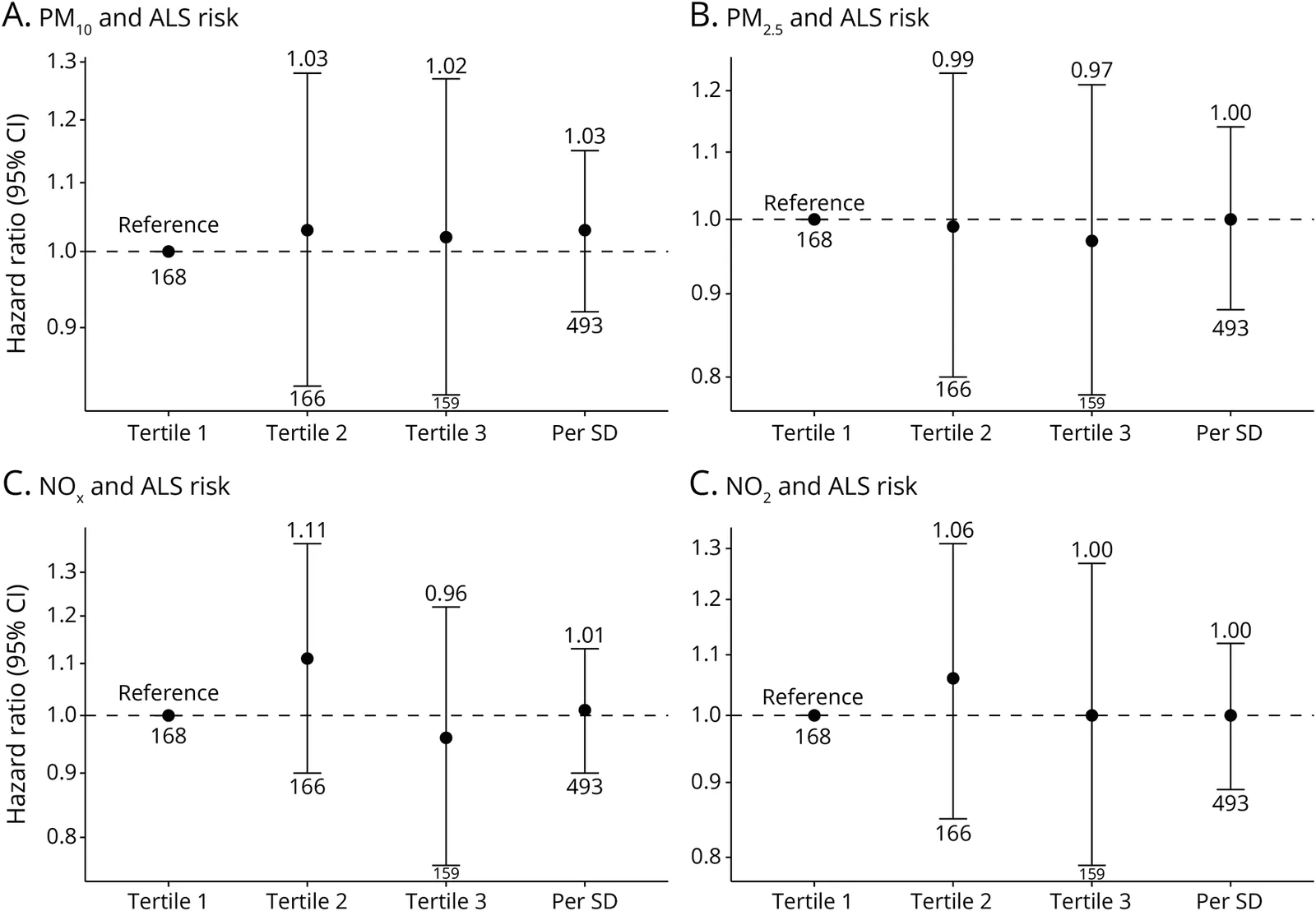
Associations Between Long-Term Exposure to Ambient Air Pollutants and ALS Risk Using Multivariable Cox Time-Varying Analysis HRs with 95% CIs for incident ALS according to tertiles and per 1 SD increase of long-term exposure to (A) PM_10_, (B) PM_2.5_, (C) NO_x_, and (D) NO_2_. The lowest exposure tertile was used as the reference category. Numbers beneath each estimate indicate the count of ALS events, while those above represent the respective HR. ALS = amyotrophic lateral sclerosis; HR = hazard ratio; NO_x_ = nitrogen oxides; NO_2_ = nitrogen dioxide; PM_10_ = particulate matter ≤10 μm; PM_2.5_ = particulate matter ≤2.5 μm.

**Figure 2:**
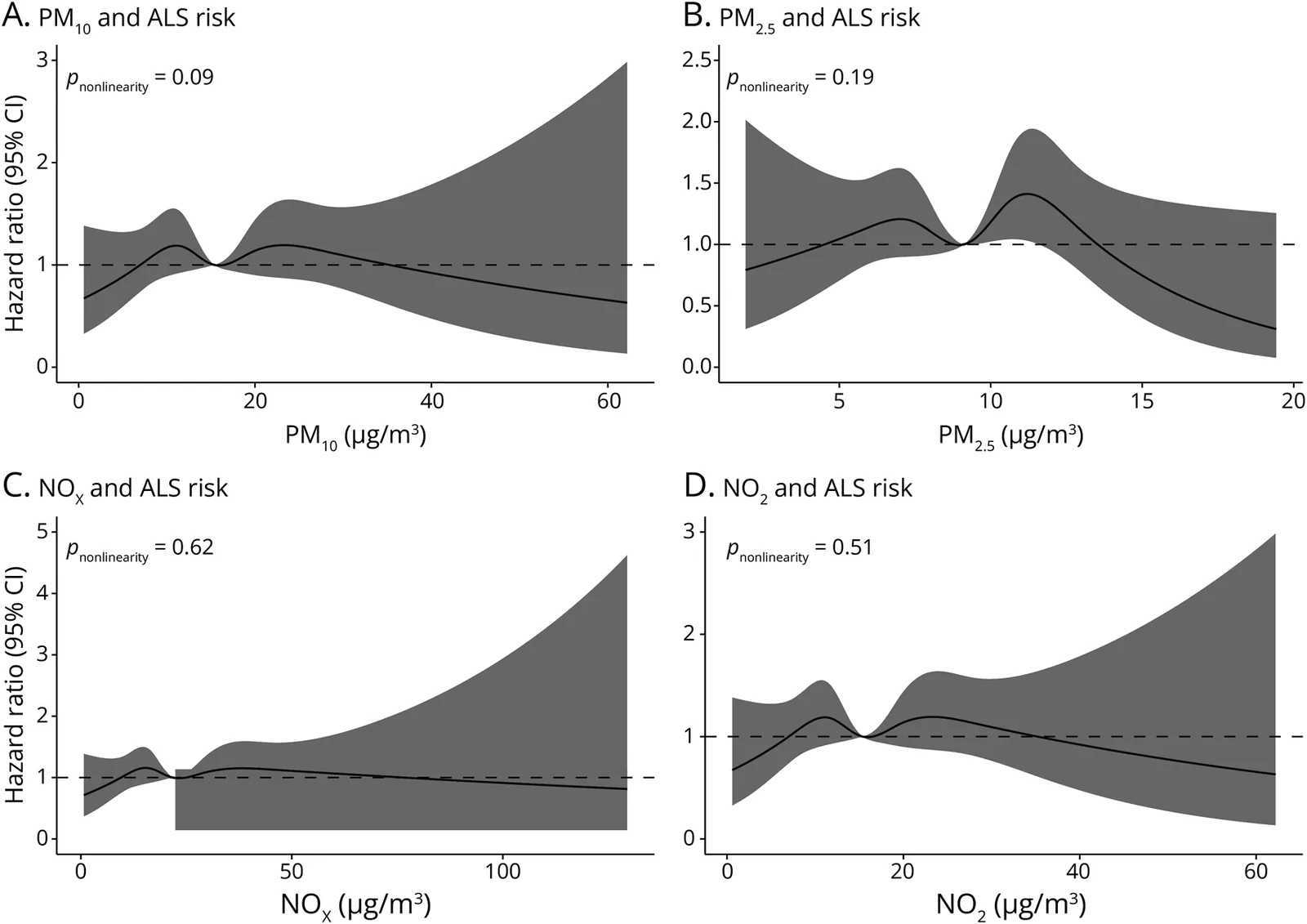
Nonlinear Association Between Long-Term Exposure to Ambient Air Pollutants and ALS Risk Using Cox Time-Varying Analysis With Restricted Cubic Splines Knots were placed at the 5th, 50th, and 95th percentiles, with the 50th (median) as the reference. ALS = amyotrophic lateral sclerosis.

Based on the power calculation, our study had ≥80% power to detect HR of approximately ≥1.06 or ≤0.94 for PM_10_ and PM_2.5_, and approximately ≥1.03 or ≤0.97 for NO_X_ and NO_2_ (eFigure 2). We did not observe any associations for any of the examined air pollutants and ALS risk. Specifically, the HRs per SD increment for PM_10_, PM_2.5_, NO_x_, and NO_2_ were 1.03 (95% CI 0.92–1.15), 1.00 (95% CI 0.88–1.14), 1.01 (95% CI 0.90–1.13), and 1.00 (95% CI 0.89–1.12), respectively (Figure 1 and eTable 3). Individuals living in areas with the highest tertile (tertile 3) of air pollutant exposure, compared with those in the lowest tertile (tertile 1), did not show a higher risk of ALS across any of the pollutants examined (*p* for trend >0.05). Although the sex-specific HRs did not individually reach statistical significance, the interaction tests for NO_X_ and NO_2_ were statistically significant (*p* = 0.026 and *p* = 0.023, respectively). These interactions indicate that the association between these pollutants and ALS risk differs by sex, with weak, nonsignificant effect estimates that are positive in men and negative in women (eTable 4). We found no evidence of interaction by *C9orf72* expansion status or by *UNC13A* rs12608932 genotype (all *p* interaction >0.05) (eTable 5). Restricted cubic spline analyses showed no nonlinear associations between air and ALS risk (all *p* nonlinearity >0.05) (Figure 2). When we restricted follow-up to different periods, re-ran the analysis including only participants who maintained the same address throughout follow-up, or used only baseline air-pollution measurements, the results remained consistent with the main analyses (eTables 6–8 and eFigure 3).

## Discussion

This study examined the association between long-term ambient air pollution and ALS incidence. With sufficient statistical power to detect even modest HRs, we found no evidence of an association between long-term exposure to ambient air pollutants (PM_10_, PM_2.5_, NO_X_, and NO_2_) and the risk of ALS. These findings were robust across multiple sensitivity analyses. We did not find any evidence of gene-environment interaction.

We focused on particulate matter and nitrogen oxides, which are established indicators of ambient air pollution. Particulate matter arises from both primary sources, such as vehicle exhaust, industrial emissions, and biomass burning, and from secondary formation in the atmosphere through reactions of gaseous precursors, including sulfur dioxide, nitrogen oxides, and volatile organic compounds. In particular, the finer PM_2.5_ fraction poses greater health risks than PM_10_ because these particles can reach the deepest regions of the lungs and enter the systemic circulation.^24^ NO_x_, primarily emitted from traffic and fossil fuel combustion, are widely used markers of urban air pollution; among them, NO_2_ is often used in epidemiologic studies as a regulated indicator and a proxy for complex mixtures of combustion by-products with potential neurotoxic effects.

Previous work in this area has yielded mixed results (summarized in eTable 1). Two previous studies using a case-control approach in the Netherlands reported associations between PM_10_, PM_coarse_, PM_2.5,_ NO_X_, and NO_2_ with ALS risk, with one of the studies also identifying an association of PM_2.5_ and ALS.^10,15^ A major limitation of this work is the uncertainty of exposure assessment. Air pollution levels were estimated using land use regression (LUR) models developed from measurements in 2009, back-extrapolated to 1992. This approach assumes that spatial contrasts in air pollution remained stable over time, an assumption that may not hold given changes in traffic intensity, fuel composition, industrial emissions, and urban planning over decades. Furthermore, pollutant concentrations between 2009 and 2013 were assumed to remain constant, despite documented downward trends in air pollution across Europe during this period. Together, these limitations highlight the potential for temporal exposure misclassification, which could bias effect estimates and complicate the interpretation of the reported associations.

Several studies have failed to show an association between airborne pollutants and ALS risk. A case-control study in Italy found an association between PM_10_ levels and ALS risk.^11^ However, the study was limited by its small sample size (52 ALS cases and 80 controls), which may have affected the precision of the estimates. Similarly, a US study found no association between exposure to metals, pesticides, organic/chlorinated solvents, and other hazardous air pollutants and ALS risk, although a significant association was found for aromatic solvents.^13^ In line with this, a study based in Denmark also reported no association between any of the examined air pollutants (long-term exposure to NOx, elemental carbon, CO, PM_2.5_, or annual O_3_) and ALS risk.^14^ Two Canadian case-control studies found no association between PM_2.5_, NO_2_, and O_3_.^25,26^

An association has been demonstrated between higher levels of SO_2_ and ALS development; however, when exposures during the 5 years preceding disease onset were excluded from the exposure window, the association reversed direction and group differences were no longer significant.^26^ We did not assess the association between SO_2_ and ALS in our study because SO_2_ levels in the United Kingdom are generally very low and model performance for SO_2_ has been poor, with validation statistics yielding R^2^ values between 0 and 0.3, and even lower in earlier years, likely reflecting concentrations near or below the detection limits of many monitors.^27^ Moreover, SO_2_ emissions in the United Kingdom are limited, arising primarily from industrial sources and shipping.

Although plausible biological mechanisms link air pollution and neurodegeneration, including oxidative stress, systemic inflammation, microglial activation, and disruption of the blood-brain barrier,^7-9^ our findings suggest that a primary effect of air pollution on these pathways does not underlie ALS pathogenesis in this cohort. Some potential caveats are nonetheless considered. First, despite the use of high-resolution exposure models, nondifferential misclassification at the individual level (e.g., due to indoor exposures, commuting patterns, or occupational environments not captured by residential models) is inevitable and would bias associations toward the null. Second, exposure levels in the United Kingdom are relatively low compared with historical or global settings, and any potential effects may lie below thresholds required to influence ALS pathogenesis. Alternatively, it is possible that although air pollution contributes to other neurodegenerative conditions, such as dementias or Parkinson disease,^4-6^ ALS may be more strongly shaped by alternative potentially modifiable or genetic risk factors.

A major strength of the UK Biobank is its prospective cohort design. All previous studies on air pollution and ALS have used a case-control design, which is more susceptible to selection bias and temporal exposure misclassification. By contrast, our cohort design minimizes these biases, ensures that exposure assessment preceded disease onset, and reduces the possibility of reverse causation. Unlike previous work that relied on back-extrapolated LUR models or limited short-term monitoring campaigns, this study uses longitudinal exposure data for air pollutants, enabling a more robust assessment of long-term environmental exposures in relation to disease risk. It is important that we were also able to account for residential changes over time, thereby capturing individual exposure histories more accurately than studies restricted to a single address.

Some limitations of this study should also be acknowledged. The well-documented selection bias in UK Biobank, favoring a healthier, wealthier, more highly educated and less ethnically diverse population than the United Kingdom in general, may affect exposure to air pollution and generability to populations with different ancestral, sociodemographic, or health profiles. Nevertheless, since the healthy volunteer effect does not affect the validity of exposure-outcome associations, our findings likely remain reliable.^28^ Another limitation relates to the method of ascertaining ALS diagnosis, which relied on hospital admission and death registry records; this approach may have led to misclassification or under-ascertainment of cases. Finally, despite adjusting for numerous covariates that could potentially affect ALS, other unmeasured or residual confounding factors might still have influenced the analysis.

In conclusion, our findings do not support a measurable effect of air pollution exposure on ALS risk in this large population-based setting, highlighting the need to investigate alternative potential environmental contributors to disease etiology.

## Glossary

ALS: amyotrophic lateral sclerosis
BMI: body mass index
DEFRA: UK Department for Environment, Food and Rural Affairs
HR: hazard ratio
HRE: hexanucleotide repeat expansion
ICD-9: International Classification of Diseases, Ninth Revision
ICD-10: International Classification of Diseases, 10th Revision
LUR: land use regression
NO_x_: nitrogen oxides
NO_2_: nitrogen dioxide
PM_2.5_: particulate matter with a diameter less than 2.5 μm
PM_10_: particulate matter with a diameter less than 10 μm
TDI: Townsend Deprivation Index
